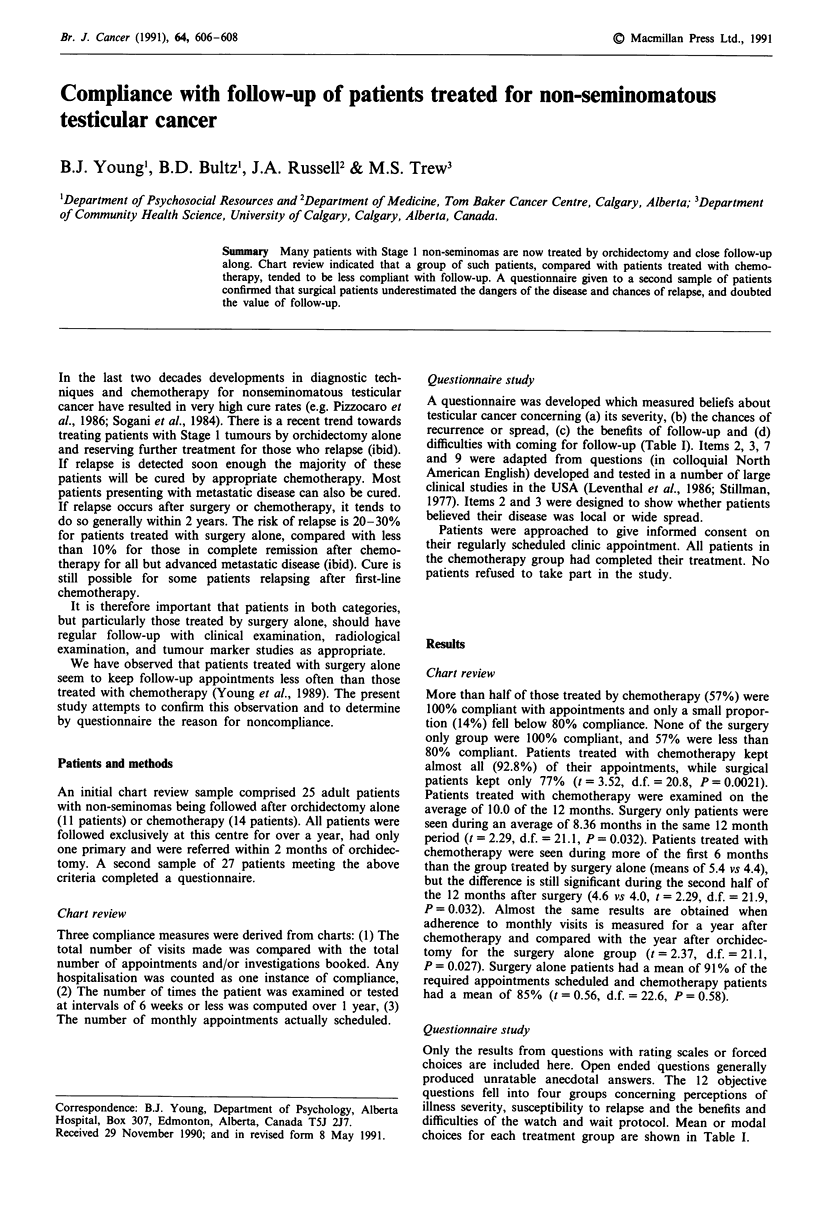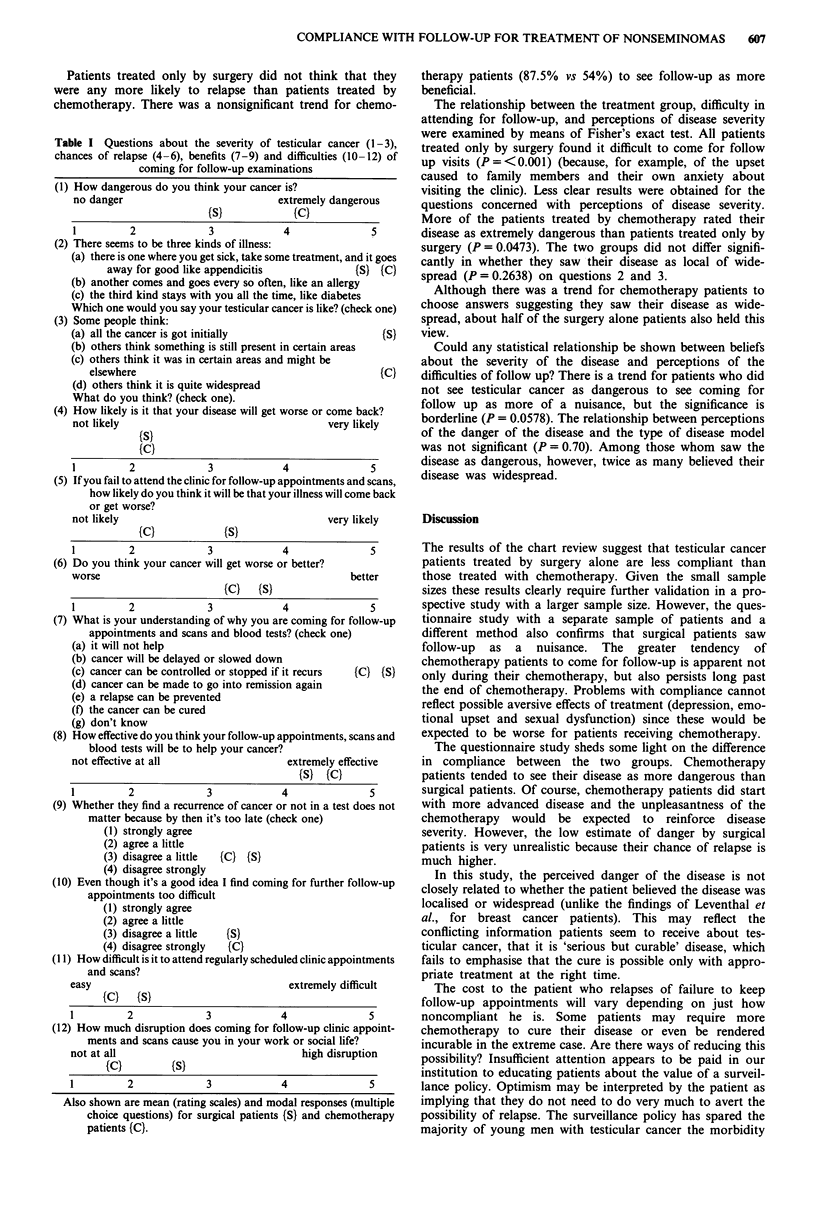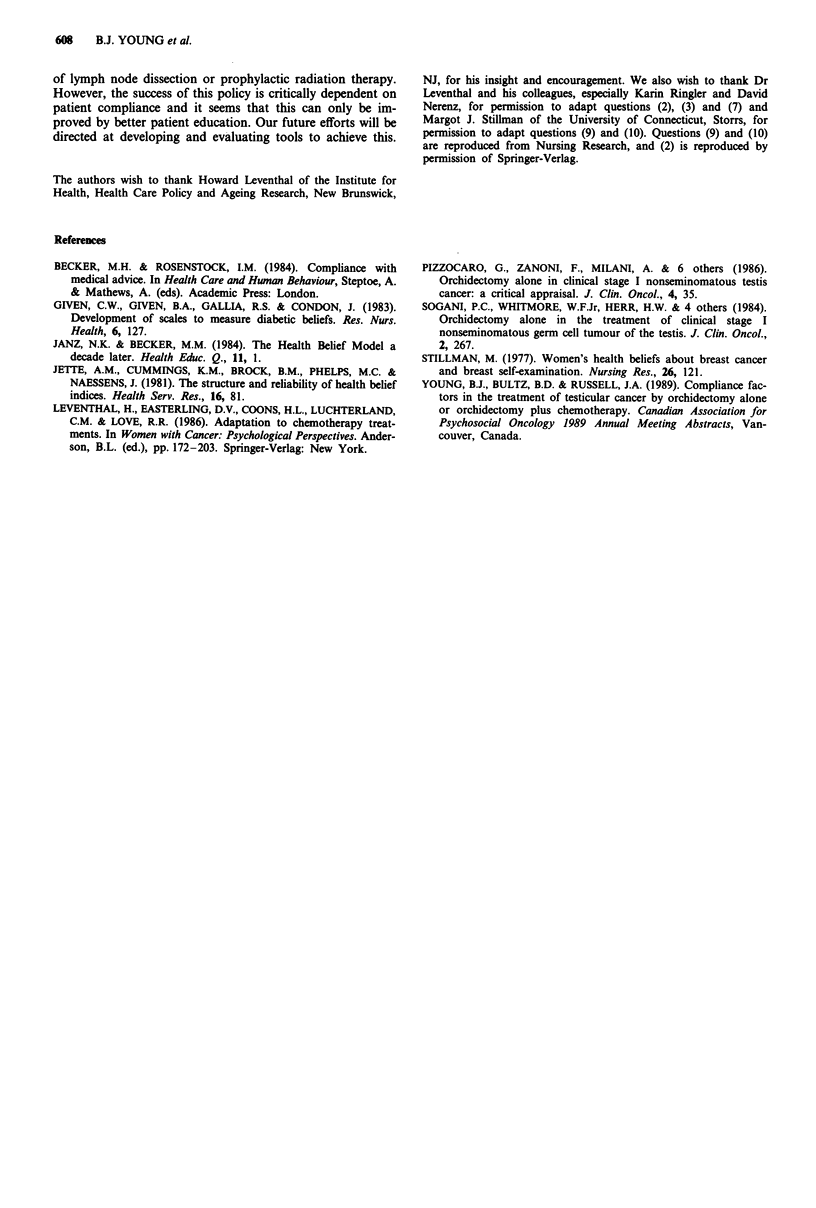# Compliance with follow-up of patients treated for non-seminomatous testicular cancer.

**DOI:** 10.1038/bjc.1991.359

**Published:** 1991-09

**Authors:** B. J. Young, B. D. Bultz, J. A. Russell, M. S. Trew

**Affiliations:** Department of Psychosocial Resources, Tom Baker Cancer Centre, Calgary, Alberta, Canada.

## Abstract

Many patients with Stage 1 non-seminomas are now treated by orchidectomy and close follow-up along. Chart review indicated that a group of such patients, compared with patients treated with chemotherapy, tended to be less compliant with follow-up. A questionnaire given to a second sample of patients confirmed that surgical patients underestimated the dangers of the disease and chances of relapse, and doubted the value of follow-up.


Br. J. Cancer (1991), 64, 606–608 © Macmillan Press Ltd., 1991

### Compliance with follow-up of patients treated for non-seminomatous testicular cancer

B.J. Young[1], B.D. Bultz[1], J.A. Russell[2] & M.S. Trew[3]

[1]*Department of Psychosocial Resources and* [2]*Department of Medicine, Tom Baker Cancer Centre, Calgary, Alberta;* [3]*Department of Community Health Science, University of Calgary, Calgary, Alberta, Canada.*

**Summary** Many patients with Stage 1 non-seminomas are now treated by orchidectomy and close follow-up along. Chart review indicated that a group of such patients, compared with patients treated with chemotherapy, tended to be less compliant with follow-up. A questionnaire given to a second sample of patients confirmed that surgical patients underestimated the dangers of the disease and chances of relapse, and doubted the value of follow-up.

In the last two decades developments in diagnostic techniques and chemotherapy for nonseminomatous testicular cancer have resulted in very high cure rates (e.g. Pizzocaro *et al.*, 1986; Sogani *et al.*, 1984). There is a recent trend towards treating patients with Stage 1 tumours by orchidectomy alone and reserving further treatment for those who relapse (ibid). If relapse is detected soon enough the majority of these patients will be cured by appropriate chemotherapy. Most patients presenting with metastatic disease can also be cured. If relapse occurs after surgery or chemotherapy, it tends to do so generally within 2 years. The risk of relapse is 20–30% for patients treated with surgery alone, compared with less than 10% for those in complete remission after chemotherapy for all but advanced metastatic disease (ibid). Cure is still possible for some patients relapsing after first-line chemotherapy.

It is therefore important that patients in both categories, but particularly those treated by surgery alone, should have regular follow-up with clinical examination, radiological examination, and tumour marker studies as appropriate.

We have observed that patients treated with surgery alone seem to keep follow-up appointments less often than those treated with chemotherapy (Young *et al.*, 1989). The present study attempts to confirm this observation and to determine by questionnaire the reason for noncompliance.

### Patients and methods

An initial chart review sample comprised 25 adult patients with non-seminomas being followed after orchidectomy alone (11 patients) or chemotherapy (14 patients). All patients were followed exclusively at this centre for over a year, had only one primary and were referred within 2 months of orchidectomy. A second sample of 27 patients meeting the above criteria completed a questionnaire.

### *Chart review*

Three compliance measures were derived from charts: (1) The total number of visits made was compared with the total number of appointments and/or investigations booked. Any hospitalisation was counted as one instance of compliance, (2) The number of times the patient was examined or tested at intervals of 6 weeks or less was computed over 1 year, (3) The number of monthly appointments actually scheduled.

### *Questionnaire study*

A questionnaire was developed which measured beliefs about testicular cancer concerning (a) its severity, (b) the chances of recurrence or spread, (c) the benefits of follow-up and (d) difficulties with coming for follow-up (Table I). Items 2, 3, 7 and 9 were adapted from questions (in colloquial North American English) developed and tested in a number of large clinical studies in the USA (Leventhal *et al.*, 1986; Stillman, 1977). Items 2 and 3 were designed to show whether patients believed their disease was local or wide spread.

Patients were approached to give informed consent on their regularly scheduled clinic appointment. All patients in the chemotherapy group had completed their treatment. No patients refused to take part in the study.

### Results

### *Chart review*

More than half of those treated by chemotherapy (57%) were 100% compliant with appointments and only a small proportion (14%) fell below 80% compliance. None of the surgery only group were 100% compliant, and 57% were less than 80% compliant. Patients treated with chemotherapy kept almost all (92.8%) of their appointments, while surgical patients kept only 77% ($t = 3.52$, d.f. = 20.8, $P = 0.0021$). Patients treated with chemotherapy were examined on the average of 10.0 of the 12 months. Surgery only patients were seen during an average of 8.36 months in the same 12 month period ($t = 2.29$, d.f. = 21.1, $P = 0.032$). Patients treated with chemotherapy were seen during more of the first 6 months than the group treated by surgery alone (means of 5.4 *vs* 4.4), but the difference is still significant during the second half of the 12 months after surgery (4.6 *vs* 4.0, $t = 2.29$, d.f. = 21.9, $P = 0.032$). Almost the same results are obtained when adherence to monthly visits is measured for a year after chemotherapy and compared with the year after orchidectomy for the surgery alone group ($t = 2.37$, d.f. = 21.1, $P = 0.027$). Surgery alone patients had a mean of 91% of the required appointments scheduled and chemotherapy patients had a mean of 85% ($t = 0.56$, d.f. = 22.6, $P = 0.58$).

### *Questionnaire study*

Only the results from questions with rating scales or forced choices are included here. Open ended questions generally produced unratable anecdotal answers. The 12 objective questions fell into four groups concerning perceptions of illness severity, susceptibility to relapse and the benefits and difficulties of the watch and wait protocol. Mean or modal choices for each treatment group are shown in Table I.

Correspondence: B.J. Young, Department of Psychology, Alberta Hospital, Box 307, Edmonton, Alberta, Canada T5J 2J7.
Received 29 November 1990; and in revised form 8 May 1991.

Patients treated only by surgery did not think that they were any more likely to relapse than patients treated by chemotherapy. There was a nonsignificant trend for chemotherapy patients (87.5% *vs* 54%) to see follow-up as more beneficial.

The relationship between the treatment group, difficulty in attending for follow-up, and perceptions of disease severity were examined by means of Fisher's exact test. All patients treated only by surgery found it difficult to come for follow up visits ($P = < 0.001$) (because, for example, of the upset caused to family members and their own anxiety about visiting the clinic). Less clear results were obtained for the questions concerned with perceptions of disease severity. More of the patients treated by chemotherapy rated their disease as extremely dangerous than patients treated only by surgery ($P = 0.0473$). The two groups did not differ significantly in whether they saw their disease as local of widespread ($P = 0.2638$) on questions 2 and 3.

Although there was a trend for chemotherapy patients to choose answers suggesting they saw their disease as widespread, about half of the surgery alone patients also held this view.

Could any statistical relationship be shown between beliefs about the severity of the disease and perceptions of the difficulties of follow up? There is a trend for patients who did not see testicular cancer as dangerous to see coming for follow up as more of a nuisance, but the significance is borderline ($P = 0.0578$). The relationship between perceptions of the danger of the disease and the type of disease model was not significant ($P = 0.70$). Among those whom saw the disease as dangerous, however, twice as many believed their disease was widespread.

**Table I** Questions about the severity of testicular cancer (1–3), chances of relapse (4–6), benefits (7–9) and difficulties (10–12) of coming for follow-up examinations

(1) How dangerous do you think your cancer is?
no danger — extremely dangerous
{S} {C}
1 2 3 4 5

(2) There seems to be three kinds of illness:
(a) there is one where you get sick, take some treatment, and it goes away for good like appendicitis {S} {C}
(b) another comes and goes every so often, like an allergy
(c) the third kind stays with you all the time, like diabetes
Which one would you say your testicular cancer is like? (check one)

(3) Some people think:
(a) all the cancer is got initially {S}
(b) others think something is still present in certain areas
(c) others think it was in certain areas and might be elsewhere {C}
(d) others think it is quite widespread
What do you think? (check one).

(4) How likely is it that your disease will get worse or come back?
not likely — very likely
{S}
{C}
1 2 3 4 5

(5) If you fail to attend the clinic for follow-up appointments and scans, how likely do you think it will be that your illness will come back or get worse?
not likely — very likely
{C} {S}
1 2 3 4 5

(6) Do you think your cancer will get worse or better?
worse — better
{C} {S}
1 2 3 4 5

(7) What is your understanding of why you are coming for follow-up appointments and scans and blood tests? (check one)
(a) it will not help
(b) cancer will be delayed or slowed down
(c) cancer can be controlled or stopped if it recurs {C} {S}
(d) cancer can be made to go into remission again
(e) a relapse can be prevented
(f) the cancer can be cured
(g) don't know

(8) How effective do you think your follow-up appointments, scans and blood tests will be to help your cancer?
not effective at all — extremely effective
{S} {C}
1 2 3 4 5

(9) Whether they find a recurrence of cancer or not in a test does not matter because by then it's too late (check one)
(1) strongly agree
(2) agree a little
(3) disagree a little {C} {S}
(4) disagree strongly

(10) Even though it's a good idea I find coming for further follow-up appointments too difficult
(1) strongly agree
(2) agree a little
(3) disagree a little {S}
(4) disagree strongly {C}

(11) How difficult is it to attend regularly scheduled clinic appointments and scans?
easy — extremely difficult
{C} {S}
1 2 3 4 5

(12) How much disruption does coming for follow-up clinic appointments and scans cause you in your work or social life?
not at all — high disruption
{C} {S}
1 2 3 4 5

Also shown are mean (rating scales) and modal responses (multiple choice questions) for surgical patients {S} and chemotherapy patients {C}.

### Discussion

The results of the chart review suggest that testicular cancer patients treated by surgery alone are less compliant than those treated with chemotherapy. Given the small sample sizes these results clearly require further validation in a prospective study with a larger sample size. However, the questionnaire study with a separate sample of patients and a different method also confirms that surgical patients saw follow-up as a nuisance. The greater tendency of chemotherapy patients to come for follow-up is apparent not only during their chemotherapy, but also persists long past the end of chemotherapy. Problems with compliance cannot reflect possible aversive effects of treatment (depression, emotional upset and sexual dysfunction) since these would be expected to be worse for patients receiving chemotherapy.

The questionnaire study sheds some light on the difference in compliance between the two groups. Chemotherapy patients tended to see their disease as more dangerous than surgical patients. Of course, chemotherapy patients did start with more advanced disease and the unpleasantness of the chemotherapy would be expected to reinforce disease severity. However, the low estimate of danger by surgical patients is very unrealistic because their chance of relapse is much higher.

In this study, the perceived danger of the disease is not closely related to whether the patient believed the disease was localised or widespread (unlike the findings of Leventhal *et al.*, for breast cancer patients). This may reflect the conflicting information patients seem to receive about testicular cancer, that it is 'serious but curable' disease, which fails to emphasise that the cure is possible only with appropriate treatment at the right time.

The cost to the patient who relapses of failure to keep follow-up appointments will vary depending on just how noncompliant he is. Some patients may require more chemotherapy to cure their disease or even be rendered incurable in the extreme case. Are there ways of reducing this possibility? Insufficient attention appears to be paid in our institution to educating patients about the value of a surveillance policy. Optimism may be interpreted by the patient as implying that they do not need to do very much to avert the possibility of relapse. The surveillance policy has spared the majority of young men with testicular cancer the morbidity of lymph node dissection or prophylactic radiation therapy. However, the success of this policy is critically dependent on patient compliance and it seems that this can only be improved by better patient education. Our future efforts will be directed at developing and evaluating tools to achieve this.

The authors wish to thank Howard Leventhal of the Institute for Health, Health Care Policy and Ageing Research, New Brunswick, NJ, for his insight and encouragement. We also wish to thank Dr Leventhal and his colleagues, especially Karin Ringler and David Nerenz, for permission to adapt questions (2), (3) and (7) and Margot J. Stillman of the University of Connecticut, Storrs, for permission to adapt questions (9) and (10). Questions (9) and (10) are reproduced from Nursing Research, and (2) is reproduced by permission of Springer-Verlag.

### References

BECKER, M.H. & ROSENSTOCK, I.M. (1984). Compliance with medical advice. In *Health Care and Human Behaviour*, Steptoe, A. & Mathews, A. (eds). Academic Press: London.

GIVEN, C.W., GIVEN, B.A., GALLIA, R.S. & CONDON, J. (1983). Development of scales to measure diabetic beliefs. *Res. Nurs. Health*, **6**, 127.

JANZ, N.K. & BECKER, M.M. (1984). The Health Belief Model a decade later. *Health Educ. Q.*, **11**, 1.

JETTE, A.M., CUMMINGS, K.M., BROCK, B.M., PHELPS, M.C. & NAESSENS, J. (1981). The structure and reliability of health belief indices. *Health Serv. Res.*, **16**, 81.

LEVENTHAL, H., EASTERLING, D.V., COONS, H.L., LUCHTERLAND, C.M. & LOVE, R.R. (1986). Adaptation to chemotherapy treatments. In *Women with Cancer: Psychological Perspectives*. Anderson, B.L. (ed.), pp. 172–203. Springer-Verlag: New York.

PIZZOCARO, G., ZANONI, F., MILANI, A. & 6 others (1986). Orchidectomy alone in clinical stage I nonseminomatous testis cancer: a critical appraisal. *J. Clin. Oncol.*, **4**, 35.

SOGANI, P.C., WHITMORE, W.F.Jr, HERR, H.W. & 4 others (1984). Orchidectomy alone in the treatment of clinical stage I nonseminomatous germ cell tumour of the testis. *J. Clin. Oncol.*, **2**, 267.

STILLMAN, M. (1977). Women's health beliefs about breast cancer and breast self-examination. *Nursing Res.*, **26**, 121.

YOUNG, B.J., BULTZ, B.D. & RUSSELL, J.A. (1989). Compliance factors in the treatment of testicular cancer by orchidectomy alone or orchidectomy plus chemotherapy. *Canadian Association for Psychosocial Oncology 1989 Annual Meeting Abstracts*, Vancouver, Canada.